# Supplementary material for: Are carbon emissions trading and green financial instruments synergistic? -Comprehensive quantitative research based on content analysis
Source: PLoS One. 2024 Mar 7;19(3):e0298601. doi: 10.1371/journal.pone.0298601 (PMC10919601; doi:10.1371/journal.pone.0298601)
Supplement: S1 Data — (ZIP) [file pone.0298601.s001.zip › Data/地级市Z_F工作报告.zip!/╦─┤¿/╥╦▒÷╩╨/└┤╫╘╣1⁄2╓┌║┼í╛┬φ┐╦╩2╛▌═°í┐_2015.docx]

各位代表：

我代表市人民政府，向大会报告过去一年的政府工作，对今年工作提出建议，请各位代表审查，并请市政协委员提出意见。

2014年工作回顾

2014年，是我市近年来经济形势最为严峻复杂、经济社会发展备受考验和挑战的一年，也是全市上下加快改革创新、各项工作取得显著成效的一年。在省委、省政府和市委的坚强领导下，在市人大及其常委会的工作监督、法律监督以及市政协的民主监督下，我们全面贯彻中央、省上和市委的决策部署，紧扣“产业转型、创新发展”的总体取向和“稳位求效、创新求进”的工作要求，大力实施“2365”发展战略，在全省率先出台“稳增长”政策及一系列配套措施，全力以赴保持了经济平稳较快发展。全市地区生产总值1443.81亿元，增长8%，总量保持全省第四位；规模以上工业增加值增长8.2%；地方公共财政收入105.6亿元，增长8.9%，总量保持全省第四位；社会消费品零售总额561.69亿元，增长13%，总量保持全省第四位；全社会固定资产投资1130.26亿元，增长20.8%，总量居全省第五位、比上年提升一位；城镇居民人均可支配收入24990元，增长10%；农民人均纯收入9831元，增长11.6%；民生持续改善，社会和谐稳定，较好地完成了市四届人大四次会议确定的年度目标任务。

一、狠抓产业转型，经济增长质量稳步提高

坚持稳增长、调结构、促转型有机结合，在千方百计稳住工业的同时加快发展现代服务业和现代农业，三次产业的协调性进一步增强。工业经济实现止滑回升。深入实施工业强市战略，着力打基础、抓重点、转观念，“三个中心、六大基地”规划全面编制完成，市（县、区）领导带队调研指导556户重点工业企业，扶持、减负等措施得到较好落实。着力引导五粮液集团等名优白酒企业调整产品结构、创新营销模式，五粮液品牌价值提升到735.8亿元，全市白酒行业发展止滑企稳。切实解决煤炭企业发展的市场、融资、税负等难题，深入推进整顿关闭和兼并重组，煤炭产业回升向好。积极推进综合能源深度开发、先进装备制造等产业发展，长宁区块页岩气产能达到2.77亿立方米，建成全国第一口自主经营高产井和第一条输气管线，率先实现工厂化生产并向居民供气。继续支持优势企业发展壮大，中核建中、惊雷科技、川茶集团经营效益持续提升。强化中小企业培育，全市新增规模以上工业企业66户。加快14个产业园区发展，实现规模以上工业总产值1422.4亿元，增长13.9%。四川长江工业园加快规划建设，五粮液产业园区国家新型工业化示范基地通过国家工信部复核。深入推进创新驱动战略，国家知识产权试点城市通过验收，国家创新型试点城市创建工作有序推进。创建国家级企业技术中心1户、省级6户、市级5户。新培育科技型中小企业21户，创建高新技术企业10户。现代农业加快发展。“米袋子”、“菜篮子”主要农产品供给充足，茶叶、蚕茧产量稳居全省第二位，粮油、林竹、畜禽、果蔬等特色优势农业稳步发展。国家农业科技园区规划建设加快实施。全市建成现代农业万亩示范区16个、农业科技试验示范基地39个、万亩林亿元钱示范区10个。服务业发展势头良好。服务业增加值完成368.01亿元，增长10%，占GDP的比重提高0.9个百分点。成功举办早茶节、川滇黔渝藏旅游产品交易会、中国白酒文化节等大型促销活动。东方时代广场、银龙广场、西南机械装备贸易城等97个服务业项目顺利实施。我市被确定进入全省7个“二级物流园区布局城市”和6个重点物流地区，宜宾港入围全国首批进境粮食指定口岸名单，集装箱吞吐量12.03万标箱，增长99%；保税物流中心（B型）基础设施基本建成。加快推进国际生态文化旅游目的地建设，旅游业总收入256.05亿元，增长22.7%。金融业健康稳定发展，全市农村信用社改制顺利完成，市商业银行第二轮增资扩股基本完成，成都银行宜宾分行、国泰君安宜宾营业部、五粮液财务公司开业运营，保险、咨询、中介、电子商务加快发展。

二、狠抓项目投资，经济发展后劲持续增强

坚持把稳投资作为稳增长的关键，深入实施项目推进战略，一大批打基础、补短板、增后劲、惠民生的重大项目加快实施。向家坝水电站全部8台机组并网发电，天竹公司竹纤维等项目竣工投运，天原集团20万吨塑胶管路等项目顺利推进，中核建中核燃料元件新厂址搬迁扩能等项目开工建设，武乐煤矿等项目前期工作进展顺利。宜宾港重大件泊位建成投运，S308线长宁县城过境公路、S309线珙县巡场过境公路建设全面完成。宜宾新机场试验段、成贵高速铁路宜宾段和宜宾至叙永高速公路、宜宾至彝良高速公路、绕城高速公路西段等项目加快实施，宜宾至屏山新县城快速通道、筠连至巡司公路、纳黔高速江门互通至兴文公路、大塔高速公路连接线等项目建设有序推进。川南城际铁路内自宜段、宜宾至威信高速公路、宜宾至攀枝花高速公路、南溪至内江高速公路以及南溪至泸州快速通道、珙县巡场至孝儿至上罗公路、宜宾县孔滩至柏溪公路前期工作进展顺利。王家沟、新坝、东山、龙滚滩、蟠龙湖、漂水岩水库等重点水利工程加快建设，向家坝灌区北总干渠一期工程前期工作顺利推进。124个省级新村基础设施建设项目全面完成。全市新建、改建通村公路2200公里。扎实推进项目策划、包装，储备2015年拟实施的项目1500余个，总投资超过5000亿元。

三、狠抓民生改善，社会大局保持和谐稳定

坚持把保障和改善民生作为政府工作的出发点和落脚点，实实在在为群众谋福祉，城乡居民的幸福感进一步提升。全年投入重点民生工程专项资金99.7亿元，“九项民生工程”及18件民生实事的各项指标全面完成。统筹推进各类重点群体就业，城镇新增就业6.87万人，城镇登记失业率为3.8%。发放青年创业小额贷款2.4亿元。不断扩大社会保险覆盖面，各类社会保险参保人数达412万人次。全面实现养老、工伤、生育、失业等社会保险市级统筹，建立统一的城乡居民基本养老保险和大病保险制度，企业职工补充保险和养老、工伤、生育等社保待遇水平进一步提高。开工保障性住房和改造棚户区住房2.54万套（户），基本建成1.28万套（户）。成功争取国开行60亿元贷款用于棚户区改造，在全省排名第一。全面实施特殊困难群众专项帮扶制度，城乡社会救助体系进一步完善。建成106个城市和农村社区日间照料中心、114个农村互助养老设施。市儿童福利院、市康复医院建成投用，市社会福利院迁建顺利推进。第九届村（居）民委员会换届选举工作全面完成。大力实施精准扶贫，全年共减少贫困人口8.65万人，向家坝移民后扶发展规划完成评审。生态市建设成绩位列全省前茅，创建国家级生态乡镇5个，江安县、南溪区顺利通过省级生态县验收。全面启动国家环境保护模范城市创建工作。完成10户企业落后产能淘汰任务，预计全市单位生产总值能耗下降5.5%。福溪电厂、珙县电厂降氮脱硝工程全面完成，与华电集团达成黄桷庄电厂先停后关的共识。中心城区环境空气质量状况总体良好，三江流域宜宾段水质达到Ⅲ类标准，全市森林覆盖率提高到43.5%。支援甘孜州雅江县和新龙县经济社会发展项目17个，投入援建资金5000万元。“4.25”双河地震灾后重建基本完成。创新社会治理，网格化服务实现全覆盖。深化“平安宜宾”建设，“多破案、严防控、惠民生”等专项整治行动有效推进。安全生产管理和监督进一步加强，安全生产形势总体稳定。强化食品药品监管，未发生重大食品安全事故。创新信访工作机制，充分发挥“市长与网民”等民意诉求平台的重要作用，一大批群众反映强烈的问题得到有效解决。应急管理、气象和防震减灾全面加强，妥善处置了“5.12”公交车燃烧等事件。大力推进国防后备力量建设，积极支持驻宜部队建设，大学生征兵入伍工作位于全省前列，国防教育、人民防空、消防、优抚、安置、交通战备等工作成效明显，国家安全、保密工作取得新成绩。

四、狠抓城乡统筹，区域发展格局更加优化

深入实施城乡统筹发展战略，加快城乡一体化，发展的协调性进一步增强。城市总体规划首次通过省政府批复，历史文化名城保护规划和14项中心城区控规、城市设计、专项规划编制完成。全面推进城镇提升改造，城乡建设投资360亿元，新增建成区面积20平方公里，城镇化率提高1.4个百分点。南部新区、岷江新区、临港新区、南溪新区、金沙新区、李庄新区、三江口等区域开发建设加快实施，环长江旅游景观大道项目有序推进。中心城区旧城改造完成投资18.75亿元，南北线提升改造顺利推进，对131条城市道路实施黑化改造，三江口滨江景观带初现雏形。全市房地产开发投资171.86亿元，增长21.8%；建筑业总产值达188.35亿元。强力开展城市管理十大治理，中心城区拆除违法建筑12.3万平方米、大型户外广告97处，加快实施城市照明设施升级改造，地下管网、农贸市场、停车场进一步完善。中心城区新增公交线路1条、公交车80辆、出租车200辆，投放免费公共自行车3910辆。市、县（区）数字城管平台初步实现互联互通。省级园林城市创建工作通过考评验收，全国卫生城市、国家级园林城市、国家森林城市创建工作有序推进，珙县成功创建为国家卫生县城，长宁县通过国家卫生县城第二轮复审。沿江经济带和山区资源综合开发区发展协调推进，地区生产总值超过百亿的县（区）新增4个、达到8个，有8个县（区）GDP增速高于全市平均水平，8个县（区）公共财政收入超过5亿元。非公有制经济加快发展，实现增加值827.75亿元，占GDP的比重达57.3%。全市完成6个“乡改镇”，12个省级“百镇建设行动”试点镇加快建设，市级试点示范小城镇行动有序推进。切实改善农村面貌，初步建成418个新村、21个新农村综合体和67个幸福美丽新村示范村。

五、狠抓改革开放，发展动力和活力不断迸发

进一步明确了全面深化改革的任务书、时间表、路线图，制定169条先行先试的改革举措，重点领域和关键环节改革加快推进。行政管理体制改革和扩权强县、扩权强镇改革进一步深化，筠连县、珙县、屏山县成为全省扩权强县试点县。新一轮政府机构改革全面完成，政府职能转变持续推进。研究出台了《关于进一步深化改革加快临港开发区发展的意见》。公共资源交易电子化和政府信息公开标准化建设全面实施。工商登记制度改革成效明显，新增企业4976户，增长48.3%。户籍制度、农村产权制度、城乡社会保障制度、用地制度和农村金融创新“五项改革”加快实施。农村产权确权颁证工作顺利推进，土地经营权全面放活。有序推进国有企业改革，深化投融资体制改革，有效落实了企业和基层投资自主权。积极推进PPP试点，在全省率先出台《关于推进政府和社会资本合作试点工作的指导意见》，在全省首批28个PPP项目签约仪式上成功签约6个，总投资26.51亿元，数量居全省首位，工作成效得到财政部和省政府充分肯定。深化资源性产品价格改革，探索资源合理利用。财税体制改革深入推进，市本级政府性债务风险保持在可控范围内。扩大“营改增”试点工作有序实施。招商引资取得新突破，全年共履约市外项目889个，市外到位资金725.4亿元，增长14.7%。在第十五届西博会上签约项目238个，签约金额1096.5亿元。区域交流合作不断深入，与长三角、珠三角等区域的合作持续加强，推动川南经济区一体化取得较好成效。新增青岛、厦门航班，直航城市达14个。对外经贸合作继续拓展，外贸进出口总额8.9亿美元，茶叶出口占全省的60%以上。外事、外侨、台事、贸促、海关、检验检疫等工作成效明显。

六、狠抓公共服务，各项社会事业全面进步

农村义务教育“四大工程”累计完成投资17.3亿元，翠屏区、南溪区县域义务教育均衡发展顺利通过新一轮国家评估认定。高考本科上线人数连续9年增长、连续5年上线超过1万人，重点本科上线人数比上年增长40%。职业教育加快发展，成立了矿山、汽车职教集团。学前教育、高等教育、特殊教育稳步发展，宜宾学院新设立了川茶学院。民办教育发展取得新成效。体育设施不断完善，全民健身活动蓬勃开展。成功承办四川省第十四届少数民族传统体育运动会，组队参加第十二届省运会并创下历史最好成绩。严格执行国家基本药物制度，县级公立医院综合改革稳步推进，医疗保障体系进一步完善，新农合参合率达99.5%。法定传染病发病率连续6年保持全省最低水平。长江国际医养中心、市二医院临港医院等项目稳步推进。第一时间驰援云南鲁甸地震灾区，圆满完成33名地震伤员在宜宾的救治任务。计划生育绩效管理控制目标顺利完成，全面落实了单独二孩政策。文化事业加快发展，市广电中心即将投入使用，一批示范乡镇文化站、幸福美丽新村（社区）文化院坝、示范农家书屋和公共电子阅报屏相继建成。启动第四届省级文明城市创建工作，新增8个全国文明村镇、单位。中心城区和县城有线电视数字化整体转换基本完成。全国质量强市示范城市通过预验收，被评为全国“年度质量魅力城市”。筠连县、珙县、屏山县被批准为少数民族地区待遇县。新闻出版、统计、电子政务、地方志、档案、住房公积金、机关事务管理等工作成效明显，民族宗教、爱国卫生、红十字、科协、工会、青少年、妇女儿童、老龄、残疾人等事业取得新成绩。

各位代表，回顾一年来的工作，我们深切的感受到，只有坚持专注发展、主动作为，才能持续推进经济增长；只有坚持改革创新、先行先试，才能牢牢把握发展主动权；只有坚持综合施策、狠抓落实，才能推进各方面工作进步；只有坚持以人为本、民生优先，才能得到群众的赞成和拥护。我们将坚持这些行之有效的做法和举措，并在工作实践中不断优化提升，推动全市经济社会发展取得更大成效。

过去一年各项成绩的取得，是全市上下在市委的坚强领导下，同心同德、团结拼搏的结果，也是各级各界关心支持的结果。在此，我代表市人民政府，向各位人大代表、政协委员，并通过你们向全市各族人民、驻宜部队、武警官兵、政法干警、各民主党派、工商联、无党派人士、各人民团体和各界朋友表示衷心的感谢，并致以崇高的敬意！

在肯定成绩的同时，我们也清醒地看到，发展中还存在不少困难和问题。受宏观经济下行压力的影响，少数经济目标未能圆满完成；科学统筹资源配置的方法还不多，传统支柱产业发展仍然面临较大困难，新的经济增长点还不稳固，产业转型升级任重道远；土地、资金、环境容量等要素的硬约束进一步加剧，一些重大招商引资项目落地困难，部分在建项目推进缓慢，投资高位增长很难持续；保障和改善民生的任务依然繁重，城乡居民增收的难度加大，住房、教育、医疗、城市交通等群众关注的热点问题亟待进一步解决。这些问题在当前和今后一段时期内将长期存在，我们将以对党和人民高度负责的态度，正视问题和差距，坚持不懈地加以改进，决不辜负全市各族人民的期望！

2015年工作安排

2015年是全面完成“十二五”规划的收官之年，也是我市率先崛起、全面建成小康的关键之年，改革发展的任务艰巨繁重。综合分析各方面的情况，我市结构调整的阵痛不断加剧，各类风险和矛盾将进一步显现，经济惯性下滑的压力仍然较大。但我们也要看到，我国进入经济发展新常态，国家推动“一带一路”发展、长江经济带建设以及省上深入实施“三大发展战略”、加快推进“两个跨越”，这些都为我市带来了新的发展机遇。我们务必要主动适应新常态，着力抢抓新机遇，更加坚强有力地推进各项工作，促进全市经济平稳健康发展和社会和谐稳定。

今年政府工作的总体要求是：全面贯彻党的十八大、十八届三中、四中全会精神，以邓小平理论、“三个代表”重要思想、科学发展观为指导，认真贯彻习近平总书记系列重要讲话精神，全面落实中央、省委经济工作会议和市委四届九次全会的工作部署，主动适应和把握经济发展新常态，围绕“产业转型、创新发展”的总体取向，坚持以提高经济发展质量和效益为中心，把转方式、调结构放到更加重要的位置，深入实施“2365”发展战略，着力内需拉动、产业带动、创新驱动，深化改革开放，强化民生保障，奋力开创经济社会发展新局面，在率先崛起、全面建成小康的道路上迈出更加坚实的步伐。

今年全市经济社会发展的主要预期目标是：地区生产总值增长8.5%左右；规模以上工业增加值增长9%左右；服务业增加值增长10.5%左右；全社会固定资产投资增长12%左右；社会消费品零售总额增长12.5%左右；地方公共财政收入增长8.5%左右；城镇化率提高2个百分点左右；城镇新增就业5.5万人以上，城镇登记失业率控制在4.2%以内；城镇居民人均可支配收入增长10%左右；农民人均纯收入增长11%左右；完成省上下达的物价调控、节能减排等目标任务。在全面完成“十二五”规划各项目标任务的同时，高标准编制完成“十三五”规划。

面对经济新常态、发展新任务、群众新期盼，推动今年经济社会发展目标圆满完成，实现有质量、有效益、可持续的发展，我们必须切实把握好四个关键。

一是要牢牢把握“产业转型、创新发展”的总体取向。坚持“2365”发展战略各项部署，加快改造提升传统优势产业，大力发展高新技术产业和战略性新兴产业，不断优化产业结构。深入实施创新驱动发展战略，加快推进经济增长由要素支撑向创新驱动、资源依赖向科技依托、宜宾制造向宜宾创造转变，不断提高经济核心竞争力。

二是要牢牢把握“深化改革、扩大开放”的发展路径。坚持把改革开放作为推动经济社会发展的根本动力，切实抓好重点领域和关键环节改革，持续激发市场主体活力。坚定不移地扩大开放，以开放促改革、增活力，积极承接产业转移，为全市经济社会发展提供强大支撑。进一步加强对外合作交流，切实推进川南经济区一体化发展。

三是要牢牢把握“依法治市、环境兴市”的基本要求。坚持用法治思维和法治方式推进政府各项工作，健全民主决策机制，依法履行政府职能，全面推进政务公开，广泛接受各方面监督，加快建设法治政府。进一步巩固提升环境兴市成果，持续转变作风，提高行政效能，全面优化经济社会发展环境。

四是要牢牢把握“改善民生、服务群众”的宗旨意识。坚持发展依靠人民、发展为了人民，切实把改善和服务民生放在更加突出的位置，持续加大民生投入，促进社会公平正义。加快推进城乡一体化发展，推动基础设施和公共服务向农村延伸，实现发展成果更多更公平惠及全市人民。

围绕全年目标任务，我们将着力抓好十个方面的工作。

一、着力加快转型升级，推动工业提质增效

扎实推进传统产业转型发展。进一步完善和出台扶持六大基地发展的政策措施。支持白酒企业调整优化产业发展模式，探索建立区域性酒业创新设计示范基地，着力提高“宜宾酒”的整体竞争力和市场占有率。大力发展煤电、煤炭深加工和瓦斯综合利用，探索发展煤化工，做大、做强、做安煤炭产业。着力培育发展一批产业龙头企业，鼓励兼并重组和战略合作，积极推动化工、轻纺等传统产业特色发展、成链发展。继续实施大企业大集团倍增计划，促进一批中小企业扩大规模和转型发展。

加快发展战略性新兴产业。积极制定实施新材料、新能源、先进装备制造、信息技术、节能环保和生物产业发展路线图。加速页岩气勘探开发和就地转化利用，力争年产气17亿立方米。加快推进中核建中核燃料元件新厂址搬迁扩能项目建设，积极打造四川惊雷科技产业园、西部磁电产业园、天原新材料产业园等战略性新兴产业示范基地。推动工业化和信息化深度融合，加快发展研发设计、检验检测、现代物流、信息服务、商务服务等生产性服务业，力争新增省级生产性服务业示范企业3户。

进一步加大科技创新。鼓励企业形成具有自主知识产权的核心技术和专利技术，积极参与国家、行业标准的制定。支持企业在关键技术、关键工艺上进行技术创新合作，实施一批重大产学研合作项目。加强科技成果推广与应用，推进园区生产力促进中心、企业技术中心、重点实验室和科技孵化园等研发平台建设，力争创建国家级企业技术中心1户、省级3户。大力实施质量强市和品牌带动战略，完成全国质量强市示范城市创建工作。

狠抓产业园区建设。围绕“园区有主业、一园一特色”，进一步优化园区产业规划与布局。切实推进园区道路、水电气、通讯等基础设施建设，健全园区配套服务体系。加快推进四川长江工业园规划建设，大力发展临港“飞地园区”，鼓励和支持有条件的县（区）跨省、市、县（区）联建园区。积极推进五粮液、惊雷科技等企业“以企建园”。

二、着力抓好项目建设，保持投资稳定增长

健全项目综合协调机制。进一步发挥好重大项目指挥部的作用，建立健全重点项目考核、进展情况月报、分级协调、督查督办、定期通报等机制。认真落实项目投资工作县（区）和市级部门行政首长负责制。积极探索建立政府投资项目公示制度。建成全市项目管理信息系统，提高项目精细化管理水平。

加快重点项目建设。深入实施“立体交通大会战”，加快推进成贵高速铁路宜宾段、宜宾机场迁建、宜宾至叙永高速公路、宜宾至彝良高速公路、绕城高速公路西段等项目建设，力争开工建设川南城际铁路内自宜段、宜宾至威信高速公路、仁沐新高速公路、宜宾至仁寿高速公路、宜宾至庆符公路、宜宾港散货码头等项目，做好宜宾至攀枝花高速公路、长江航道宜宾段Ⅲ级升Ⅱ级改造提升等项目前期工作。突出抓好产业项目建设，开工建设嘉业机械整体搬迁等35个项目，竣工投产鸿源化工60万吨硫酸等46个项目，确保工业投资370亿元以上。切实完善现代水利设施，加快向家坝灌区南北首部取水隧洞和北总干渠一期工程及新坝、蟠龙湖、龙滚滩、王家沟、东山水库等项目建设。

加强项目包装与储备。认真研究国家政策和资金投向，做好规划、政策和项目对接，争取更多项目列入国家、省上的规划和投资计划。紧紧围绕“三个中心、六大基地”建设，进一步做深做细项目论证和储备，着力策划一批带动力强、影响面广、事关经济社会发展的重大项目，动态充实项目储备库。

三、着力增进民生福祉，全面发展社会事业

切实抓好就业和社会保障工作。认真实施“九项民生工程”。积极推进人社信息化建设，加快实现县乡村人社网点和信息系统全覆盖。切实加强劳动保障监察信息网络化、监管网格化建设。继续做好高校毕业生、农村转移劳动力、城镇困难人员、退役军人等重点群体就业工作。完善社会保险制度，推进机关事业单位养老保险制度改革和城乡居民医保整合。切实加强社会保险基金监管。大力开展特殊困难群众救助帮扶工作。加快发展养老事业，鼓励支持社会力量发展养老服务业。实施好保障性安居工程建设，新开工15054套，基本建成8227套，竣工7480套，发放公租房租赁补贴8409户。

优先发展教育事业。进一步深化教育综合改革，全面推进素质教育，持续提升教育教学质量。加强教师队伍建设，完善义务教育学校教师“县管校聘”机制。加快推进普通高中特色化和差异化改革。着力发展现代职业教育，加快推进宜宾职业技术学院国家级骨干高职院校建设。实施第二轮学前教育三年行动计划，进一步发展特殊教育、高等教育和继续教育，积极推动宜宾学院向应用型综合大学转型发展。规范有序发展民办教育。加大投入力度，全面完成农村义务教育“四大工程”建设任务，加快实施薄弱义务教育学校改造工程。不断优化学校校点布局，抓好校园安全和周边环境整治。

加快卫生事业发展。深化县级公立医院综合改革，巩固基本药物制度成果。巩固新农合覆盖水平，加快推进新农合信息化建设。积极建立基层首诊、双向转诊、分级医疗、上下联动的医疗服务新模式。切实加强乡村医生队伍建设。加快推进长江国际医养中心、市妇幼保健院迁建、市二医院临港医院等项目建设。继续加强重点传染病的防控。积极发展健康服务业。认真落实计划生育利益导向优惠政策和单独二孩政策。

扎实推动文化体育等事业发展。继续深化公益性和经营性事业单位改革。大力发展文化产业，积极组建宜宾日报报业集团、宜宾广电集团。完成市广电中心建设，开工建设市博物馆新馆。完成全市有线电视数字化整体转换工作。着力实施文化惠民工程，建设65个幸福美丽新村（社区）文化院坝、46个市级示范农家书屋和50个公共电子阅报屏。积极加强文化遗产保护工作。深入实施全民健身计划，启动奥运金牌十年计划，开工建设宜宾体育活动中心。扎实推进市社会福利院二期工程建设。开工建设市政务服务中心、市残疾人综合服务中心。积极稳妥做好行政区划调整工作。新建农村社区20个。继续抓好民族宗教、地方志、爱国卫生、红十字、电子政务、住房公积金、机关事务管理、科协、档案、工会、青少年、妇女儿童、老龄、残疾人等事业发展。

四、着力发展现代服务业，积极扩大市场消费

全面提升商贸物流业水平。加快推进东方时代广场、重庆新世纪百货、西南机械装备贸易城等项目建设，积极培育创建美食城、美食街和特色商业街，鼓励和支持县（区）打造富有特色的次级商圈。支持唐人财富中心、莱茵春天等城市商业综合体提升商业业态，抓好叙府商城、燕君连锁、绿源食品、西南轻工博览城、临港汽车产业园等重点商贸流通企业培育和发展。切实加快临港国际物流园区和象鼻、金沙、五粮液等物流中心发展，加强农产品冷链、城市物流配送等物流基础设施建设。积极推动长江经济带海关通关一体化改革，力争保税物流中心（B型）获批投用。重点培育一批龙头物流企业，促进现代物流与制造业、商贸业、电子商务联动发展。

加快发展壮大旅游业。着力推进旅游基础设施建设，进一步完善“吃、住、行、游、购、娱”要素配套。做好旅游公共信息服务，加快智慧旅游建设。推动旅游与文化融合发展，大力发展文化生态旅游、工业旅游、城市旅游、乡村旅游，培育多层次旅游业态。全力承办好2015中国•四川国际文化旅游节。加快推进国家5A级、4A级景区创建提升工作，积极打造国家级和省级生态旅游示范区、旅游度假区、休闲农业与乡村旅游示范县，加快建设国际生态文化旅游目的地。推动组建川南旅游产业战略联盟，加强旅游跨区域合作。

培育壮大现代金融业。着力完善金融业发展的扶持政策，加快推进川南城市群金融中心建设。继续深化银政企交流合作。进一步做好企业上市工作，鼓励企业利用多层次资本市场加快发展。积极引进各类金融机构，重点推进光大银行、民生银行、乐山市商业银行入驻宜宾。继续抓好金融生态环境建设，切实防范和化解金融风险。

积极培育消费热点。深入开展“扩内需、促消费”系列活动，大力促进节假日消费。组织参加全国、全省重大展会，不断拓展产品销售渠道。努力搭建产销对接平台，认真举办好第二届采购商大会、第八届中国宜宾早茶节、2015中国白酒文化节、第三届宜宾特色农产品展示展销活动、第六届豆腐干博览会。推动电子商务等新兴先导型服务业发展，加快五粮液以及茶叶等农产品电子商务平台建设。大力发展信息消费、健康养老消费。

五、着力创新农业经营方式，促进农民持续增收

大力发展特色优势产业。进一步调整优化农业产业结构和农产品品种结构，大力发展茶叶、牛羊、水产等“741”特色优势产业。加快推进国家农业科技园区建设。扎实推进农业标准化示范基地建设，着力打造10个现代农业万亩示范区、45个农业科技试验示范基地和10个万亩林亿元钱示范区。积极推进省级现代农业、林业、畜牧业重点县建设。加快构建现代农业产业体系、新型农业经营体系和社会化服务体系。着力解决土地撂荒问题。提高农业机械化水平。大力引导、支持农村土地流转，加快发展农业适度规模经营。

加快建设绿色食品精深加工基地。发展壮大农产品加工园区，努力形成农业产业化、集群化发展格局。培育壮大新型农业经营主体，新增市级以上龙头企业15户、年营销额1000万元以上的农民合作社10个、家庭农场300个。切实抓好“三品一标”农产品认证登记保护工作。完成市、县级农产品质量安全追溯监管平台建设和全市50家农产品生产单位追溯信息管理平台建设。

千方百计增加农民收入。切实优化农民增收结构，多渠道促进农民增收。大力扶持发展农村持续稳定增收项目，鼓励农民通过创业增加家庭经营性收入。扎实开展就业技能培训，引导农民通过就地就近就业增加工资性收入。全面推进农村产权制度改革，支持农民通过拓展投资领域增加财产性收入。落实强农惠农政策，加大政府扶持力度增加农民转移性收入。

全面推进新农村建设。以人的新农村建设为核心，坚持产业先行、产村相融，统筹推进省级新农村示范县建设。全面完成3个省级新农村建设示范县、8个市级新农村示范片三年规划目标任务。加强农村公路、水利、农机、电力、广电等基础设施建设，实施宽带乡村惠民工程，加快建设“1+N”村级公共服务中心。加快幸福美丽新村建设，建成262个新村、12个新农村综合体和96个幸福美丽新村。加快农村廉租房建设，切实解决一批农村困难群众住房问题。

六、着力推进新型城镇化，建设现代宜居城市

全面加快新型城镇化进程。力争新增建成区面积17平方公里，城乡建设投资突破360亿元。以人的城镇化为核心，进一步深化户籍制度、土地管理制度改革，推动城乡一体化发展。完善“全域宜宾”规划体系，完成历史文化名城保护规划报批，积极推进各区域详细规划及相关专项规划编制工作。切实抓好小城镇建设，深入实施全省“百镇建设行动”，重点培育和发展中小城镇，实现省、市级重点镇投资4.3亿元。修订完善城市园林绿化相关制度及标准，切实抓好国家园林城市创建工作，开展好园林县城、城镇、单位、小区、新村创建工作。

继续抓好中心城区建设。加快推进临港新区、南部新区、岷江新区、金沙新区、三江口南岸区域、南溪新区、李庄新区等区域开发建设，确保中心城区新增建成区8平方公里，完成投资100亿元。继续推进环长江旅游景观大道、南北线提升改造及中心城区路面黑化工程，全力实施南部新区七星城公共设施和金沙城骨干道路工程建设。加快推进城市规划馆、市民中心、市工会“两中心”、南广高架和大溪口城市广场等重点项目开工建设，启动新机场连接线、天池滨江路、现代有轨电车示范线、国际会展中心等项目工作。积极推动蜀南大道和叙府路交通节点、戎州大桥南桥头交通节点改造工程等项目建设，加快构建市区立体交通体系，不断完善城市功能。合理引导住房消费，促进房地产市场健康发展。

切实提升城市管理水平。加快推进“智慧城市”建设。全面抓好数字化城市管理。完善城市管理“月考”制度。加快推动垃圾焚烧发电、垃圾处理厂渗滤液技改等项目建设。深化城市管理十大专项治理，着力解决车辆乱停、摊点乱摆、房屋乱建、广告乱象等问题。实施全域创卫工程，确保中心城区创建国家卫生城市通过省级考核，积极争创中国人居环境范例奖。

七、着力加强区域统筹，壮大县域经济实力

加快推动县域经济发展。县域经济是我市适应新常态，推动产业转型、创新发展和经济可持续发展的主战场。要继续实施交通、农田水利、生产生活环境三大提升工程，不断改善县域发展条件。优先发展沿江经济带，加快构建沿江产业群、城镇群和港口群，不断提升产业集中度和综合竞争力。启动全市长江经济带、岷江经济带产业转型发展规划编制，大力发展生态产业文明。加快发展山区资源综合开发区，促进资源综合开发利用、就地转化和直接富民。因地制宜发展壮大一批工业镇、商贸镇、旅游镇，努力形成县域经济次中心。全面落实好少数民族地区待遇县政策，推动少数民族地区、边远山区、革命老区加快发展。

大力发展非公有制经济。实行“负面清单管理”，进一步放宽社会资本进入基础设施、公用事业等行业和领域。切实帮助非公企业解决融资、用地等困难。鼓励组建和完善各种行业性商会（协会）。积极推动优质企业股份制改造，建立健全中小企业政策支持体系，实施中小微企业成长工程，培育一批创新能力强、成长速度快、市场竞争力强的“小巨人”企业。

扎实抓好精准扶贫和移民后扶工作。坚持区域发展与精准扶贫相结合，大力实施乌蒙山片区扶贫规划。积极统筹政府、市场、社会等资源，加大财政专项扶贫投入力度，整合行业扶贫、社会扶贫和移民后扶力量，切实抓好“六大扶贫工程”，减少贫困人口6.52万人。切实落实好向家坝移民后扶发展规划，不断提高移民生产生活水平，确保移民移得出、稳得住、逐步能致富。

健全区域协调发展机制。切实推进简政放权，从审批权限、项目安排、信贷投入、能源调度、人才管理等方面给予县（区）更多的自主权。加大以市带县力度，坚持因地制宜、分类指导，完善经济考核指标体系和办法。建立健全县（区）之间的沟通合作机制和协调发展机制，引导县（区）资源共享、园区共建、产业共融。

八、着力深化改革开放，增强发展动力活力

全面推进重点领域改革。深入推进统筹城乡综合配套改革，深化扩权强县、扩权强镇改革，建立统筹城乡发展机制。全面深化农村改革，加快推进农村产权制度改革。深化国有企业改革，大力推动产权多元化，鼓励社会资本积极参与国有企业改制重组。推进投融资体制改革，大力推进PPP试点，激发和释放民间投资活力。进一步推进财税体制改革。加强政府性债务管理，健全政府性债务风险预警机制。深化文化、医疗卫生体制改革。完善促进就业创业的体制机制，形成合理有序的收入分配格局。

加强对外开放和区域合作。积极推进与成渝经济区、攀西资源开发区等区域城市和友好城市的交流合作，加强与环渤海、长三角、泛珠三角的区域合作。积极加强与法国、德国、俄罗斯、东盟等国家和地区的交流合作。继续加大对重点外贸企业的扶持，支持白酒、茶叶、丝绸、竹制品等特色产品出口。支持企业到境外投资，加强外派劳务市场的监督管理。

努力提升招商引资实效。围绕“三个中心、六大基地”产业规划，加快制定投资促进产业指导目录。进一步完善招商项目科学规划方案和工作推动方案，不断创新招商形式，大力开展驻点招商、小分队招商和以商招商，尽快引进一批世界500强、国内500强、央企和省内知名大企业。强化项目跟踪服务与对接，推动签约项目尽快落地。

扎实推动临港开发区改革发展。进一步创新体制机制，完善配套政策，着力先行先试、创新发展。围绕打造产业港、创新港、开放港，重点抓好11个功能片区建设。切实加大开放合作力度，探索与沿海国家级开发区、先进地区合作共建产业园区。依托宜宾港大力推进长江起点航运中心建设，不断做大港口物流、交通、信息、产业平台，力争集装箱吞吐量达到15万标箱。

九、着力建设生态文明，加快打造美丽宜宾

切实抓好节能减排。大力实施节能改造、节能技术产业化示范等重点工程。严格控制“两高一资”和产能过剩行业新上项目。加快产业结构调整和落后产能淘汰步伐，积极推进黄桷庄电厂关闭相关工作。全面完成“十二五”污染减排目标，强化环境容量保障工作。健全减排约束机制，进一步落实减排责任，严格环境准入，严控排污总量。加快推进市、县（区）城市污水处理厂二期工程及配套管网建设，提升城市污水处理能力。

全面加强环境保护。积极推进国家环保模范城市创建工作。切实加强建设项目环评和“三同时”管理。强化环境监管执法，推进环境监察、监测、信息监控机构标准化建设。进一步提升工业集中区环境风险防控能力。强力推进大气污染防治行动计划，加强区域联防联控，狠抓工业大气污染、城市扬尘污染、餐饮油烟和机动车尾气治理，减少雾霾天气。强化向家坝库区生态环境保护。加强小流域污染综合整治，保护和改善“三江九河”水环境质量。深入推进农村面源污染、土壤环境保护和综合治理。

积极推进生态建设。制定和完善生态文明建设考核指标体系，守住生态红线，扩大生态绿线。完成高县、筠连县、珙县、兴文县省级生态县建设任务，加强生态乡镇、生态村等“生态细胞”建设。继续推进国家森林城市创建工作，确保成功创建为省级森林城市。巩固并扩大天然林保护、退耕还林成果，加强水土流失综合治理。

十、着力营造良好环境，保持社会和谐稳定

切实提升服务水平。最大限度地放宽工商注册登记条件，扩大市场主体总量。严厉打击商业贿赂和扰乱市场经济秩序、企业生产经营秩序等违法行为。规范执法工作流程，依法细化、量化自由裁量权。实行执法检查预告、检查登记等制度，杜绝随意检查、重复检查和多头检查。严禁以任何名义对企业乱收费、乱摊派、乱罚款、乱培训。加强监督管理，推动与行政审批有关的中介机构规范运作。

强化生产要素保障。建立健全“要素资源跟着项目走”的要素保障机制，突出抓好资金、土地、环境、人才等要素保障。积极争取地方留存电、留存气等扶持政策，着力抓好煤电油气运等要素的综合协调。积极组织企业参加各类产销对接活动，帮助企业开拓市场。认真落实重点项目审批“绿色通道”制度，促进项目尽快落地。加强企业家队伍培育，积极帮助企业解决生产、技术、管理、品牌、信息等方面的困难。

提升社会治理能力。深化“平安宜宾”、“法治宜宾”建设，依法严厉打击各类违法犯罪活动，加强社会治安综合治理。扎实开展法治示范创建活动，加强全民普法教育。引导群众依法反映诉求，大力开展信访案件清理化解，落实涉法涉诉信访依法终结制度。认真开展重大决策社会稳定风险评估。深化城乡社区治理体制创新，全面推进网格化服务管理。大力推进社会组织监管改革，支持和发展志愿服务组织。完善安全监管责任体系，突出抓好重点行业领域安全综合整治，促进全市安全生产形势持续稳定好转。强化食品药品安全监管。加强社会信用体系建设。完善防灾减灾和应急救援体系，做好地震、测绘、气象、水文工作。加强国家安全和保密工作。

加强国防建设。大力推进全国双拥模范城“四连冠”创建工作。积极做好国防教育、国防动员、人民防空、交通战备、优抚安置、消防等工作，进一步抓好征兵和民兵预备役建设。

加强法治政府建设

各位代表！过去的一年，我们大力推进政府自身建设，努力打造法治政府、服务政府、效能政府、廉洁政府，各项工作都取得了较好成绩。我们以整风精神开展党的群众路线教育实践活动，大力整改8个方面58个细项的问题，制定实施了精文简会、基层调研活动管理、提高议事决策执行效率等70余个制度。我们认真落实依法治市各项任务，坚持市政府常务会学法，加强行政决策风险评估和合法性审查，获得第三届“中国法治政府奖”提名奖。我们深入推进环境兴市战略，全面推行并联审批，行政审批项目从216项减少到91项，减少58%，成为全省保留行政审批项目最少的市州之一。办理行政复议案件375件。积极开展行政调解工作，市本级受理行政调解案件11068件，成功调解10533件，成功率达95%。建立基层法律援助工作站点195个，无偿办理法律援助案件3953件。认真办理人大代表建议意见383件，政协委员提案意见287件，办复率达100%。我们切实加强重大问题调研和政府目标绩效管理，政府系统全市性会议同比减少64.4%，大力整治“庸懒散浮拖”以及不作为、慢作为、乱作为，对34个单位、53名责任人进行了问责。我们进一步加大监察、审计力度，深化正风肃纪，坚决抵制各类不良作风，查处“四风”案件152件、通报曝光49起、处分110人，查处违反中央八项规定案件70起、处理80人，干部清正、政府清廉的形象进一步树立。

我们也清醒地认识到，与上级的要求和群众的期盼相比，政府自身建设还有一些差距。比如，部分决策部署和工作措施还没有完全落实到位，少数部门的行政效能和工作人员的办事效率还不够高，依法行政和反腐倡廉建设还需要加强。新的一年，我们将认真践行党的群众路线，切实解决存在的问题，着力提高推动科学发展的能力和水平，努力开创法治政府建设新局面。

切实加强依法行政。认真贯彻中央依法治国、省委依法治省和市委依法治市的要求，切实落实依法行政的主体责任，大力营造学法、用法、守法、护法、畏法的社会氛围。全面落实重大行政决策法定程序，建立行政决策终身责任追究制度及责任倒查机制。健全政府常务会议和部门办公会议学法制度。加强行政复议工作。坚决贯彻执行市人大及其常委会的决议决定，加强与市政协的沟通联系，认真办理人大代表议案、建议和政协委员提案、意见，推进办理结果公开。主动接受党内监督、人大监督、政协和民主党派监督、司法监督、社会监督、舆论监督。

切实优化政务环境。继续深化环境兴市战略，强化政务公开和各领域办事公开，建立并公开政府部门权力清单，逐项梳理行政许可、行政审批、行政处罚等事项。加大“两集中、两到位”工作力度，简化办事程序。增强服务意识，建立市县乡三级并联审批服务，探索建立网上办理和电话办理机制。充分发挥12345市民服务平台作用，加强市县乡村一体化服务体系建设，“一站式”办理群众行政许可、信访诉求、民生投诉等便民事项。

切实提升行政效能。认真贯彻“三严三实”要求，进一步强化政府班子建设，加强对干部的教育管理，大力整治不作为、慢作为、乱作为以及“庸懒散浮拖”等机关病，努力建设一支高素质的干部队伍。全面落实首问责任制、限时办结制和责任追究制，促进行政机关高效履职。按照权力和责任相适应的原则，盯住不落实的事，追究不落实的人，让权力行使者有职有责、任职负责、失职问责成为一种常态。

切实强化廉政建设。坚决贯彻从严治党、从严治政各项规定，认真落实党风廉政建设责任制，扎实推进政府系统惩治和预防腐败体系建设。坚决贯彻中央八项规定和国务院“约法三章”，稳妥推进公务用车制度改革，强化办公用房管理，继续严控“三公”经费和会议费。加强行政权力行使监督，对公共资金、国有资产、国有资源和领导干部经济责任情况实行审计全覆盖。加强行政监察，深化正风肃纪专项治理，纠正行业不正之风。严肃查处腐败案件，始终保持惩治腐败的高压态势。

各位代表！做好今年的各项工作，任务艰巨，使命光荣。让我们紧密地团结在以习近平同志为总书记的党中央周围，在省委、省政府和市委的坚强领导下，在市人大、市政协的监督支持下，团结带领全市各族人民，进一步解放思想、改革创新，真抓实干、砥砺奋进，为加快实现率先崛起、全面建成小康社会的宏伟目标而努力奋斗！
